# Supplementary material for: Environmental Effects of the COVID-19 Pandemic: The Experience of Bogotá, 2020
Source: Int J Environ Res Public Health. 2022 May 23;19(10):6350. doi: 10.3390/ijerph19106350 (PMC9141921; doi:10.3390/ijerph19106350)
Supplement: Supplementary file 1 [file ijerph-19-06350-s001.zip › ijerph-1673884-supplementary.pdf]

Supplementary Material

Supplementary Table S1. Variables to be analyzed and information sources

Environmental Matrix	Variable	Data source	Period of time
**Solid Waste**	Conventional solid waste (Tons/month) generated in Bogota	Superintendency of Residential Public Service	January 2019-December 2020
	Bio-sanitary hospital waste - Personal Protective Equipment produced in Bogota (kg/month)	Special Administrative Unit of Public Utilities -UAESP-.	January 2019-December 2020
**Air Quality**	Concentration of criteria air pollutants (carbon monoxide (CO), nitrogen oxides (NO2), particulate matter less than 10 microns (PM10) and fine particulate matter (PM2.5))	Air quality monitoring network of Bogota - District Secretary of the Environment.	January 2019-December 2020
	Perception of change in air quality	Citizen characterization survey. ITHACA Project* National Institute of Health.	February 2019-May 2021
**Water Resource**	Bogotá river water quality parameters (Organic matter,ammonioum,dissolved oxygen,manganese,turbidity and conductivity)	Bogota Water and Sewer System	Maximum values in 2019 and 2020

**Transport**	Change in the choice of mode of transport used	Citizen characterization survey. ITHACA Project National Institute of Health.	February 2019-May 2021
	Use of Bogota's Rapid Transit System (BRT)	Company providing services: Transmilenio S.A.	January 2019-December 2020

Supplementary Table S2. Types of generators according to their production in kilograms per day and the frequency of waste collection.

Acronym	Type of Generator	Daily production (Kg/day)	Collection frequencies (days)
LP	Large Producer	> 100	Daily (6/7 or 7/7)
MP	Medium Producer	≥ 25 - < 100	Three times a week (3/6)
PP	Small Producer	≥ 3 - < 25	Twice a week (2/6)
MA	Microproducer A		Once a week (1/6)
MB	Microproducer B	< 3	Twice a month
MC	Microproducer C		Once a month

Supplementary Table S3. Sociodemographic characteristics of the survey participants ITHACA.

Variable		Ovelall	Female
	15-20	140	86 (61.4%)
	21-30	603	394 (65.3%)
	31-40	497	303 (61%)
Age	41-50	292	172 (58.9%)
	51-60	134	61 (45.5%)
	61-70	28	5 (17.9%)
	Missing	127	50 (39.4%)

Date of the survey	Pandemic	738	370 (50.1%)
	Pre Pandemic	1083	701 (64.7%)
Socioeconomic Strata	1	19	13 (68.4%)
	2	298	184 (61.7%)
	3	771	484 (62.8%)
	4	476	263 (55.3%)
	5	147	87 (59.2%)
	6	60	40 (66.7%)
	Missing	50	- (%)
Occupation	Unemployed	24	20 (83.3%)
	Student	422	269 (63.7%)
	Other	5	2 (40%)
	Employee	1319	780 (59.1%)
	Missing	51	0%
Educational level	High School	257	166 (64.6%)
	Doctorate	43	23 (53.5%)
	Specialization	446	268 (60.1%)
	Master	264	167 (63.3%)
	Post-doctorate	5	5 (100%)
	Undergraduate	544	318 (58.5%)
	Technicians	211	123 (58.3%)
	Missing	51	1 (2%)
Mode of transport	Walking	113	68 (60.2%)
	Bike	189	71 (37.6%)
	Bus	159	115 (72.3%)
	Vehicle	208	113 (54.3%)
	Motorcycle	89	34 (38.2%)
	Skaterboard	2	2 (100%)
	Zonal Bus	413	273 (66.1%)
	Taxi	82	60 (73.2%)
	BRT Bus	515	317 (61.6%)
	Missing	51	18 (35.3%)

Supplementary Table S4. U-Mann Whitney test to total solid waste and biosanitary waste.

Variable	Units	2019	2020	Estimated	p-value
Total Solid Waste	Ton	203751	188713.6	111	<0.05
Biosanitary Waste	Ton	975414.9	1063097.8	51	0.24

Supplementary Table S5. U-Mann Whitney and t-test to Air Quality data.

Variable	Units	2019	2020	Estimated	p-value
CO	ppm	0,9	0,5	11.343	1,82E-13**
NO_2	ppm	38,6	16,8	982	3,41E-13**
PM_{10}	µg m^{-3}	33,8	22,4	828	1,01E-05**
$PM_{2.5}$	µg m^{-3}	17,5	13,6	697	0.01253

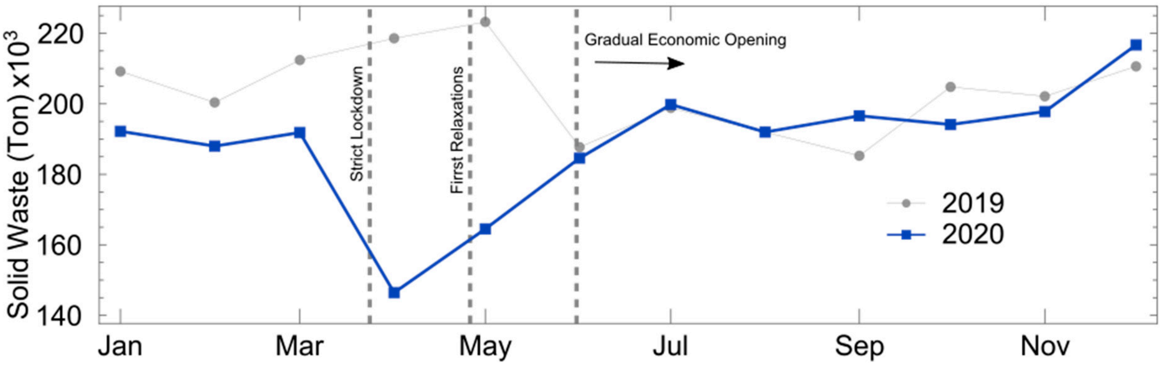

Supplementary Figure S1. Solid waste disposal series at the Bogota sanitary landfill. The gray line with circular markers indicates the tons per month for 2019, the blue line with square markers indicates the tons disposed of in 2020, the year of the pandemic.

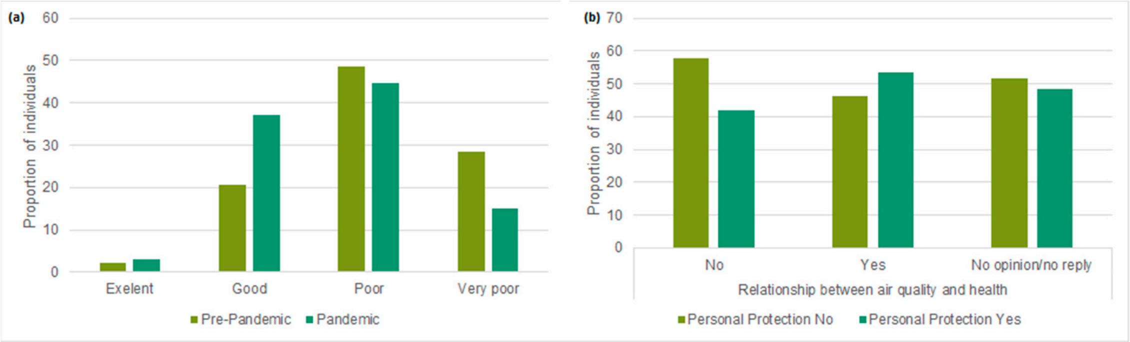

Supplementary Figure S2. Air quality perception: a) the air quality on house from job. b) Only pandemic answers about the existence of a relationship between air quality and usage of personal protection

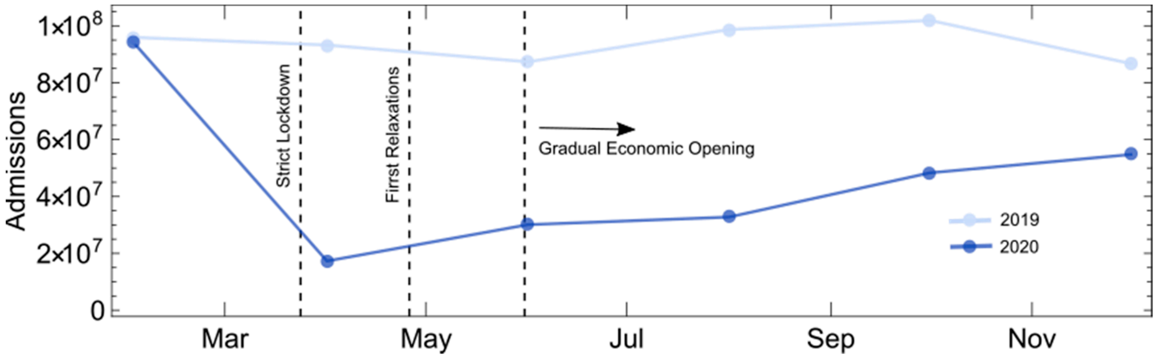

Supplementary Figure S3. BRT system admissions timeline. The light blue series shows moving concentrations in 2019 and the dark blue series shows concentrations in 2020.

– 2020.

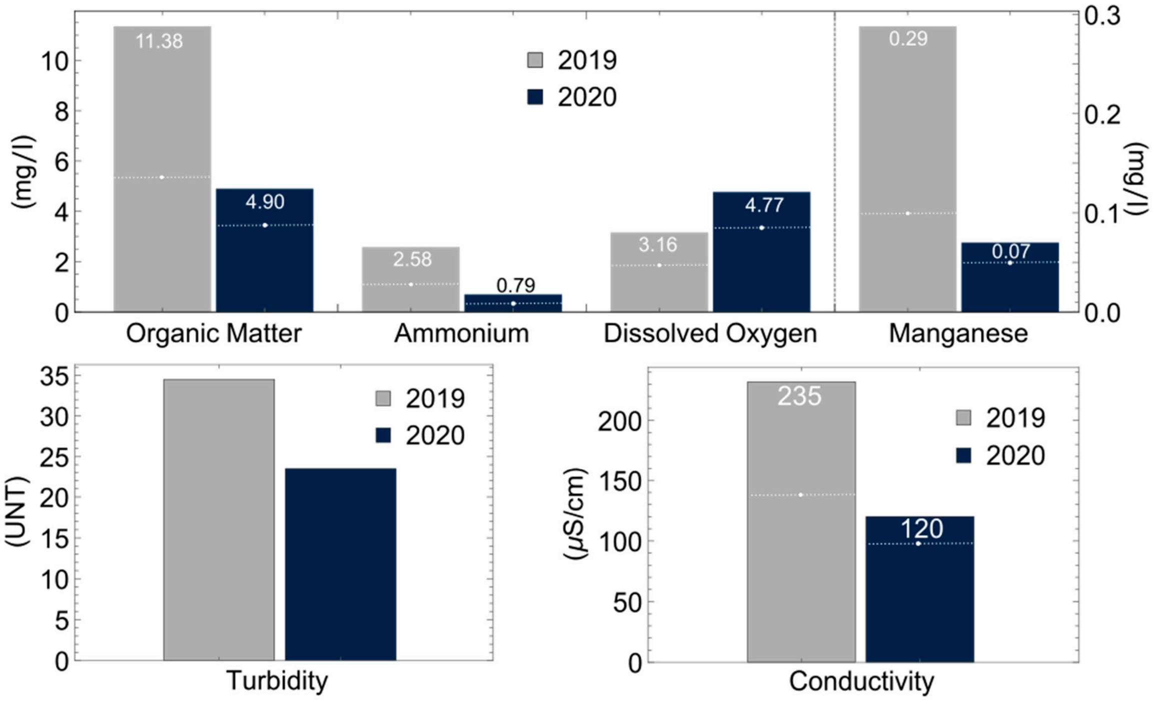

Supplementary Figure S4. Water Quality reported Bogota Water and Sewer Service 2019
